# ANP/GC-A signaling attenuates tissue injury by cisplatin

**DOI:** 10.1186/2050-6511-14-S1-P51

**Published:** 2013-08-29

**Authors:** Takashi Nojiri, Hiroshi Hosoda, Toru Kimura, Kenji Kangawa

**Affiliations:** 1 Department of Biochemistry, National Cerebral and Cardiovascular Center Research Institute, Suita, Osaka, Japan; 2 Department of General Thoracic Surgery, Osaka University Graduate School of Medicine, Suita, Osaka, Japan

## Background

Cytotoxic chemotherapy is the standard treatment in most cancer patients with advanced stage. However, many patients suffer from severe side effects even if the anti-cancer treatment is effective. There is no established strategy to prevent wide range of side effects of cytotoxic chemotherapy including appetite loss, myelosuppression, and acute renal injury.

Atrial natriuretic peptide (ANP) has been used clinically for the treatment with heart failure in Japan, and exhibits a wide range of biological activities including cardiovascular and renal protection through binding the guanylate cyclase-A (GC-A) receptor. Recent studies also reported that exogenous ANP pre-treatment reduced renal fibrosis in acute renal injury model. However, there is no evidence about ANP protection against tissue injury by cisplatin, which is a representative cytotoxic agent for most cancer treatment. This study was designed to examine whether ANP pre-treatment attenuates tissue injury including acute renal failure induced by cisplatin.

## Materials and methods

We used C57/B6 mice which were pre-treated with saline or ANP (subcutaneously via osmotic-pump, 0.5μg/kg/min) and administered intravenously a dose of 12 mg/kg cisplatin as the experimental acute renal failure mode. ANP infusion was started one day before the administration of cisplatin. This dose does not change blood pressure and heart rate in mice.

## Results

Mice with cisplatin showed weight loss, myelosuppression (decrease of white blood cell and platelet), and increased inflammatory cytokine mRNA levels in the kidney compared to the control mice. ANP-pretreated mice showed the attenuation of weight loss (19.9±0.5 vs. 15.9±0.6 g, P<0.01), myelosuppression (white blood cell; 2700±632 vs. 1300±141 cell/µl, P<0.01 , platelet; 64.8±15.5 vs. 39.6±5.7 ×10^4^ cell/µl, P<0.01), and increased tumor-necrosis factor alpha (TNFα), interleukin-6 (IL-6), and monocyte chemotactic protein-1 (MCP-1) mRNA levels in the kidney compared to the control mice with cisplatin.

## Conclusion

We found that ANP reduced the great variety of side effects induced by cisplatin. These protective effects on tissue injury induced by cisplatin was considered through anti-inflammatory effects of ANP. Our data provide novel insights into the prophylactic therapy for various side effects induced by cytotoxic chemotherapy in many cancer patients.

